# Understanding Host–Virus Interactions: Assessment of Innate Immune Responses in *Mastomys natalensis* Cells after Arenavirus Infection

**DOI:** 10.3390/v14091986

**Published:** 2022-09-08

**Authors:** Nele Marie Brinkmann, Chris Hoffmann, Stephanie Wurr, Elisa Pallasch, Julia Hinzmann, Eleonore Ostermann, Wolfram Brune, Maria Elisabeth Eskes, Lukas Jungblut, Stephan Günther, Ludmilla Unrau, Lisa Oestereich

**Affiliations:** 1 Department of Virology, Bernhard Nocht Institute for Tropical Medicine, 20359 Hamburg, Germany; 2 German Center for Infectious Disease, Partner Site Hamburg-Lübeck-Borstel-Riems, 20359 Hamburg, Germany; 3 Leibniz Institute of Virology, 20359 Hamburg, Germany

**Keywords:** arenavirus, Lassa virus, *Mastomys natalensis*, natural reservoir animal, innate immune response, interferon type I response, multiplex RT-PCR assay

## Abstract

*Mastomys natalensis* is the natural host of various arenaviruses, including the human-pathogenic Lassa virus. Homologous arenaviruses, defined here as those having *M. natalensis* as a natural host, can establish long-lasting infection in *M. natalensis,* while these animals rapidly clear arenaviruses having another rodent species as a natural host (heterologous viruses). Little is known about the mechanisms behind the underlying arenavirus–host barriers. The innate immune system, particularly the type I interferon (IFN) response, might play a role. In this study, we developed and validated RT-PCR assays to analyse the expression of *M. natalensis* interferon-stimulated genes (ISGs). We then used these assays to study if homologous and heterologous viruses induce different IFN responses in *M. natalensis* cells. Infection experiments were performed with the homologous Lassa and Morogoro viruses and the related but heterologous Mobala virus. Compared to the direct induction with IFN or Poly(I:C), arenaviruses generally induced a weak IFN response. However, the ISG-expression profiles of homologous and heterologous viruses were similar. Our data indicate that, at least in *M. natalensis* cells, the IFN system is not a major factor in the virus–host barrier for arenaviruses. Our system provides a valuable tool for future in vivo investigation of arenavirus host restrictions at the level of the innate immune response.

## 1. Introduction

Emerging viral infections pose a serious threat, and zoonotic viruses such as Nipah, Ebola, Marburg, and Lassa virus (LASV) have caused numerous human outbreaks in recent years [1,2]. The current SARS-CoV-2 pandemic also demonstrates the impact emerging viruses can have on all aspects of public health, with more than 600,500,000 people being infected and 6,472,000 deaths to date (https://covid19.who.int/, accessed on 28 August 2022). It is estimated that 75% of emerging infectious diseases in humans have a zoonotic origin [3,4,5]. The most common virus reservoirs are domestic or domesticated livestock, wildlife, and arthropods [6,7]. High population densities and poor sanitary conditions in many parts of the world, a decline in the natural habitats of many animals, climate change, and urbanisation have led to increasing human contact with animals capable of transmitting novel viruses. A better understanding of the intrinsic “virus–host–environment relationships” is key to predicting and preventing emerging or re-emerging viral diseases.

LASV, a highly pathogenic virus with pandemic potential, is a negative-stranded RNA virus of the *Arenaviridae* family that causes haemorrhagic fever in humans. LASV is endemic to West Africa and causes Lassa fever (LF), a febrile illness with symptoms ranging from mild and flu-like to haemorrhagic fever. Based on seroprevalence studies, it is estimated that up to 500,000 people become infected annually, and 5000 infected individuals dying from the disease [8]. There is currently no approved vaccine or specific treatment for humans. LASV is endemic in several West African countries, with the highest prevalence in Guinea, Liberia, Nigeria, and Sierra Leone [9], and it poses a major burden to the societies and economies of affected countries. In 2016, the World Health Organization initiated the Research and Development Plan for Epidemic Prevention Interventions. LASV is among the eight priority pathogens that pose a significant health risk and require further research and development of vaccines and therapeutics [10].

The natural host animal of LASV is the multimammate rat *Mastomys natalensis*, which is widely distributed in sub-Saharan Africa [8,11]. More recently, it has also been found in rodent species such as *M. erythroleucus, Mus minutoides, Hylomyscus pamfi,* and *Lemniscomys striatus* [12,13,14]. The underlying mechanisms that allow LASV to replicate and be transmitted in its natural host without causing disease and the factors that restrict host switching remain poorly understood. The closely related, non-pathogenic arenavirus Morogoro virus (MORV) has *M. natalensis* as a host (homologous virus), and Mobala virus (MOBV) has been isolated from *Praomys* sp. (heterologous virus) [15,16]. Infection experiments with *M. natalensis* showed that the homologous MORV could establish long-lasting infection and was transmitted between individuals. The heterologous MOBV was rapidly cleared and failed to develop long-lasting infection [13], indicating intrinsic barriers for arenavirus–host switching.

One mechanism restricting arenaviruses to their host might be the innate immune system, especially the type I interferon (IFN) response, which is known to play an important antiviral role at the early stages of viral infection [17,18,19]. The induction of the IFN system results in the expression and secretion of type I IFNs such as IFN-α, IFN-β and other proinflammatory cytokines [20]. IFNs bind to the IFN-α/β receptor on the cell surface, activating the downstream signalling pathway [21]. This activation results in the expression of interferon-stimulated genes (ISGs), which function as antiviral effectors and contribute to controlling viral replication [22]. ISGs are thought to target almost every step in the viral life cycle, such as the inhibition of viral entry (myxovirus resistance (Mx) proteins A and B), viral translation and replication (protein kinase R (PKR), interferon-induced proteins with tetratricopeptide repeats (IFIT2), zinc-finger antiviral protein (ZAP) and proteins of the 2′-5′-oligoadenylate synthetase (OAS)-RNase-L-pathway) as well as viral egress (Bone marrow stromal cell antigen 2 (BST2)). Another group of ISGs includes IRF-1 and IRF-7, which directly trigger the production of further IFN-α or IFN-β. In addition, ISGs with RNA-editing functions have been described, such as adenosine deaminase (ADAR1), which reduces the virus’s overall infectivity due to translation errors [23].

Patients infected with chronic hepatitis B virus have since more than 30 years been treated with pegylated IFN-β, which induces innate antiviral defences and thus greatly reduces the viral burden [24]. The type I IFN response has also been investigated for therapeutic intervention for highly pathogenic RNA viruses. IFN-β-1a has successfully been used to treat Ebola virus disease patients [25], and several clinical studies investigated the benefit of IFN treatment in SARS-CoV-2, MERS and SARS-infected patients [26]. Due to the high incidence of drug side effects, the usage of IFNs for treatment remains however controversially discussed. Arenaviruses, similar to many other RNA viruses, are highly susceptible to IFN-α- and -β-induced immune responses and have developed several mechanisms to suppress the activation of the IFN system. One essential protein is the viral nucleoprotein NP, which can degrade double-stranded RNA, preventing recognition by pattern recognition receptors (PRR) such as retinoic acid-inducible gene I (RIG-I) [27,28,29]. In addition, NP can directly block the translocation of IRF-3 into the nucleus [30,31] or inhibit IRF-3 phosphorylation by binding to the factor IκB kinase-related kinase IKKε, thereby blocking the transcription of IFN-α and -β mRNA [32]. The NPs of the pathogenic arenaviruses such as LASV or Junin virus have a higher suppression capacity compared to closely related but non-pathogenic viruses such as Mopeia virus (MOPV) or Tacaribe virus [31,33]. Circumvention of the IFN system is also essential for replication of LASV in mice [34,35]. Moreover, studies in non-human primates have shown that survival is linked to the development of a robust IFN response [36]. While the importance of the suppression of the IFN response on pathogenesis has been demonstrated in model organisms, it remains unclear if it is also a major factor restricting host ranges and preventing spillover into different species.

As *M. natalensis* are not a classical rodent research model, specific reagents and tools are unavailable, making immunological studies in these animals difficult. Cross-reactive antibodies against mouse, rat, or human proteins recognizing *M. natalensis* homologs are challenging to identify and validate. Research of virus–host interactions in bats, another non-classical animal model, especially relevant for many highly pathogenic viruses, primarily focuses on analysing mRNA levels to overcome the challenge of a lack of specific reagents [37]. We decided on a similar approach to assess the importance of the type I IFN response for arenavirus infection and host restriction in *M. natalensis*. In this study, we developed and validated RT-PCR assays to quantify ISG expression that can be used in in vivo studies.

## 2. Materials and Methods

### 2.1. Ethics Statement and Animals

The study was carried out in strict compliance with the recommendations of the German Society for Laboratory Animal Science under the supervision of a veterinarian. All protocols were approved by the Committee on the Ethics of Animal Experiments of the City of Hamburg (approval number O42/2018/1305/591 00 33). All efforts were made to minimise the number of animals used and to mitigate suffering during experimental procedures. All staff members involved in animal experiments and handling underwent the necessary education and training according to the Federation of European Laboratory Animal Science Associations category B or C.

*M. natalensis* were derived from our breeding colony at the Bernhard Nocht Institute for Tropical Medicine (BNITM). All animals descend from breeding pairs provided by Heinz Feldmann from the Rocky Mountain Laboratories, Montana, based on an initial colony from wild-caught arenavirus-free animals from Mali. Animals were housed in small groups in individually ventilated cages. Food and water were accessible ad libitum. Animals were euthanised by isoflurane overdose, followed by decapitation for terminal kidney or bone marrow sampling.

### 2.2. Cell Lines

Vero 76 (American tissue culture collection; ATCC, Manassas, USA) were used for virus cultivation, and quantification and cultured in DMEM supplied with 3% FCS, 1% penicillin/streptomycin/glutamine, and 2% non-essential amino acids and pyruvate. Cells were kept at 37 °C and 5% CO_2_.

To isolate *M. natalensis* kidney epithelial cells (MasKECs), one adult *M. natalensis* was anaesthetised with isoflurane and sacrificed by cervical dislocation. Kidneys were collected, decapsulated, and the medulla removed. The cortical tissue was minced and transferred to 10 mL DMEM-F12 containing 1 mg/mL Collagenase-II (Sigma-Aldrich, St. Louis, MO, USA). Cortices were then incubated at 37 °C for 20 min with vigorous vortexing every 1 min. The kidney suspension was washed with wash buffer (HBSS containing 0.2 µM HEPES, 0.45 µg/mL NaHCO₃, 80 ng/mL NaOH, 50 µg/mL Gentamycin and 2% FCS) through a metal sieve followed by one wash through a 70 µm cell strainer and two washes through a 40 µm cell strainer. Cell fragments were collected and spun down at 150× *g* for 10 min. The cell pellet was resuspended in culture medium (DMEM/F-12 containing 1× insulin/transferrin/selenium solution (Invitrogen, Waltham, USA), 2% FCS, 100 U/mL penicillin, 100 µg/mL streptomycin, 40 ng/mL hydrocortisone (Sigma-Aldrich, St. Louis, MO, USA), 0.25 nM Triiodo-L-thyronine (Sigma-Aldrich, St. Louis, MO, USA)), seeded on 1% gelatin-coated plates and incubated at 37 °C with 5% CO_2_. Culture media were replaced initially at 24 h and subsequently every 48–72 h. Once MasKECs were confluent, they were immortalised by retroviral transduction of the SV40 Large T Antigen (Addgene, Watertown, NY, USA, plasmid #14088) and kept in culture in DMEM containing 10% FCS, 100 U/mL penicillin, and 100 µg/mL streptomycin at 37 °C and 5% CO_2_.

### 2.3. Preparation of Bone Marrow-Derived Macrophage-Like Cells (MΦ)

Adult *M.* *natalensis* (>12 weeks of age) were euthanised by isoflurane anaesthesia and decapitation. The hind limbs were removed, the femur and tibia were prepared, and the bones were rinsed with 70% ethanol followed by phosphate-buffered saline (PBS) under sterile conditions. The ends of the bones were cut off with sterile scissors, and the bone marrow was flushed out with 5–10 mL sterile PBS. A single cell suspension was prepared by gently mixing and passing the cells through a 70 µm cell strainer. Cells were pelleted by centrifugation (5 min, 500× *g*) and washed with PBS. To remove all erythrocytes, the cells were resuspended in red blood cell lysis buffer (BD Pharm Lyse™; Franklin Lakes, MO, USA) and incubated for 10 min at room temperature. The lysis was stopped by adding 30 mL PBS and afterwards pelleted (5 min at 500× *g*). The cells were washed with 50 mL PBS, resuspended in 10 mL RPMI-1640 without any supplements and counted. The cells were then either seeded in six-well plates with 1 × 10^6^ cells/well in RPMI-1640 supplied with 10% FCS, 1% penicillin/streptomycin/glutamine, and 2% non-essential amino acids and pyruvate and grown at 37 °C and 5% CO_2_ or aliquoted (1 × 10^7^ cells/mL), resuspended in FCS containing 10% dimethyl sulfoxide (DMSO), frozen in a slow freezing device for cells and stored at −80 °C for up to 21 days.

Bone marrow cells were differentiated into macrophage-like cells (MΦ) by supplementing the growth media with 50 ng/mL murine M-CSF (BioLegend, San Diego, CA, USA) on the day of seeding. On day three, an equal volume of fresh RPMI-1640 containing 50 ng/mL M-CSF was added. The cell differentiation was completed six days after isolation, and the cells were used for experiments for the next two weeks. The macrophage-like phenotype was confirmed by light microscopy.

### 2.4. In vitro Stimulation Experiments

MasKECs (1 × 10^6^ cells/plate) or MΦ (1.2 × 10^7^ cells/plate) were seeded in 6-well plates and stimulated for 16 h with recombinant type I IFN (mouse IFN-α; carrier-free (BioLegend, San Diego, CA, USA) or mouse IFN-β (R&D Systems; Minneapolis, MN, USA)) in descending concentrations (1000 U/mL, 500 U/mL, 100 U/mL, and 50 U/mL).

MΦ were stimulated with 1 µg/mL Poly(I:C)-HMW/Lyovec (InvivoGen, San Diego, CA, USA) for 2, 4, 6.5, 16 and 24 h. Poly(I:C) was prepared and stored according to the manufacturer’s instructions.

### 2.5. Infection Experiments

For infection experiments, the LASV Ba366 [38], Chikungunya virus (CHIKV) Martinique strain, and the BNI-ZH501 strain of Rift valley fever virus (RVFV) [39] were used under BSL-3 and BSL-4 conditions, respectively. The MORV strain 3017/2004 isolated at the BNITM [16], and the MOBV strain 3099 [15], which was obtained from a collaborating laboratory, were handled under BSL-2 conditions. All viruses were grown on Vero 76 cells and passaged less than three times at the BNITM. Virus titres for LASV, MORV, and MOBV were determined by immunofocus assay [34] or by RT-PCR [40,41], as described previously.

For infection experiments, 1 × 10^6^ cells/well of MΦ were differentiated as described above. The cells were infected with a multiplicity of infection (MOI) of 0.1, or 1 for 1 h at 37 °C in RPMI-1640 containing 2% FCS and the corresponding amount of virus stock. After the inoculation, the cells were supplied with fresh medium and further incubated for 24–72 h at 37 °C and 5% CO_2_. The cell culture supernatant was harvested after the corresponding incubation time and stored at −20 °C (BSL-2 pathogens) or −80 °C (BSL-4 pathogens), and the cells were used for RNA extraction.

Infection experiments after in vitro stimulation of 1 × 10^6^ cells/well of MΦ with either 500 U/mL IFN-α, IFN-β, or 1 µg/mL Poly(I:C) for 16 h were conducted with LASV. Cells were infected with an MOI of 0.1 for 1 h at 37 °C. After the infection, the RPMI supplemented with the stimulants was re-applied to the cells, and they were incubated at 37 °C for 24 or 48 h.

### 2.6. Cellular RNA Isolation

After the stimulation or infection, the cell culture supernatants were removed and, in the case of infection experiments, stored at −80 °C for virus quantification by RT-PCR or immunofocus assay. The cells were rinsed with PBS, inactivated (in case of infected cells), and lysed by adding 350 µL of buffer RLT from the Qiagen RNeasy kit (QIAGEN, Venlo, Netherlands) [42]. The cells were disrupted by centrifugation through QIAShredder columns (QIAGEN, Venlo, Netherlands) according to the manufacturer’s instructions. Samples were further processed under BSL-2 conditions. The RNA was isolated according to the instructions of the RNeasy kit. The RNA was eluted in 100 µL, and the nucleic acid concentration was determined by spectrophotometric analysis with a NanoDrop (Thermo Scientific, Waltham, MA, USA). Cellular DNA contaminations were removed by treatment with TURBO^TM^ DNase (Thermo Scientific, Waltham, MA, USA) according to the manufacturer’s instructions. Purification with the Clean and Concentrator 5 kit (Zymo Research, Irvine, CA, USA), according to the manufacturer’s instructions, was performed to remove the TURBO^TM^ DNase. RNA was eluted in 15 µL elution buffer and quantified with a NanoDrop. Short-term storage of RNA was at −20 °C.

### 2.7. RT-PCR-Based Detection of Interferon-Stimulated Gene Expression

Based on previously performed transcriptome analysis of *M. natalensis* (unpublished, performed by Beijing Genomics Institute Group (BGI)) and subsequent alignment to *M. coucha* (bioproject accession numbers PRJNA578167 and PRJNA406979) [43], human and mouse transcriptomes, the coding sequences for the 60S ribosomal protein L13a (60S) and the peptidylprolyl isomerase like 4 (Ppil4) as housekeeping genes as well as the following interferon-stimulated genes were identified: ADAR1, interferon alpha-inducible protein 27B (IF27B), IFIT2, IRF1 and −7, interferon-stimulated exonuclease gene 20 (ISG20), MxA and MxB, OAS1b and OAS2, PKR as well as ZAP. Primers and probes were designed with the PrimerQuest™ Tool by Integrated DNA Technologies (www.eu.idtdna.com/PrimerQuest/Home, accessed on 18 August 2019) and synthesised by Integrated DNA Technologies, Inc. The sequences for the amplicons with primer and probe binding regions are given in Appendix A. Their respective primer and probe sequences, as well as the fluorophores and quenchers, are shown in Table S1. The two housekeeping genes 60S and Ppil4 were used as a reference for the RT-PCR-based assays.

Two to four genes with different fluorophores (FAM, Cy5, TexasRed (TxRed), and JOE) were combined per reaction, and respective colour compensations were measured on the LightCycler 480 (Roche, Basel, Switzerland). ISG expression was determined by RT-PCR with the SuperScript™III Platinum™ Taq OneStep qRT-PCR kit (Thermo Scientific, Waltham, MA, USA) based on the manufacturer’s instructions. The reaction volume was adjusted to a total of 12.5 µL, 200 nM of the primers, 100 nM of the probes and 10 ng of template RNA. All mixes used the cycling program shown in Table 1.

The fold change of expression of ISGs was calculated using the ΔΔCt method [44] with either 60S or Ppil4 as housekeeping genes and uninfected/unstimulated control cells. In short, the ΔCt value of the controls was calculated by subtracting the mean of the Ct values for the housekeeping genes from the mean of the stimulated wells or the control wells. To calculate the experimental ΔCt value, the Ct from the housekeeping gene was subtracted from the one for the ISG. The ΔΔCt value represents the difference between the experimental and control ΔCt value, whereas the expression fold change is 2^ΔΔCt^ value.

Primer efficiencies for the different genes were determined by measuring an RNA dilution series (2-fold dilution from 1:10–1:160) in a single-target RT-PCR experiment. The calculation was performed with the following equation, which describes the PCR amplification with T*_n_* being the number of target molecules at a cycle n, T_0_ as the number of target molecules at the beginning, *n* as the number of amplification cycles, and E as the amplification efficiency.

T*_n_ =* T_0_ × E*_n_*

To determine the limit of detection with a 50% detection rate (LOD_50_), RNA was diluted in a half-logarithmic dilution series (from 10^−1^ − 10^−8^) and measured in a single-target RT-PCR experiment. The Ct value corresponding to the LOD_50_ was determined by Cumulative Gaussian (to determine cut-off dilution) and sigmoidal 4PL (to determine Ct value for dilution) fitting. For both experiments, either RNA from unstimulated MΦs or MΦs stimulated with Poly(I:C) was used.

### 2.8. Software and Statistics

Data presentation, statistical analysis and plot preparation were performed in GraphPad Prism 9 (GraphPad Software, San Diego, CA, USA) for macOS (Version 9.3.1). Differences in expression levels and virus titres were calculated with one-way ANOVA and Bonferroni’s multiple comparison test. Significant differences are indicated by *p* < 0.0332 (*), *p* < 0.002 (**), *p* < 0.001 (***).

## 3. Results

### 3.1. Development and Characterisation of the RT-PCR Assays to Detect Changes in the ISG Expression

The newly designed primers were first tested in a conventional RT-PCR to verify the amplification of the correct gene via sequencing. RNA isolation, reverse transcription, and DNA depletion were optimized, and different qRT-PCR kits and PCR cyclers were tested with singleplex reactions. RT-PCR reactions without the initial reverse transcription step were performed to analyse if the extracted RNA was contaminated with DNA. No amplification was observed without the RT step. The optimized setup was used to establish two- to four-colour multiplex mixes (Table S1) for the 14 genes analysed in this study. Assessment of the two potential housekeeping gene candidates, 60S and Ppil4, showed stable expression levels in stimulated and infected cells.

Primer efficiencies for the housekeeping genes and the ISGs were in the range of 1.7–3.0 (Table 2). For IRF7, MxA, MxB, and PKR, it was not possible to determine the primer efficiency with RNA from unstimulated cells, and RNA from Poly(I:C) stimulated MΦ was used. The Ct values corresponding with the limit of detection of the assays were determined by serial dilution of RNA from stimulated cells.

### 3.2. In Vitro Stimulation Experiments to Detect Changes in ISG Expression

**Figure 1:**
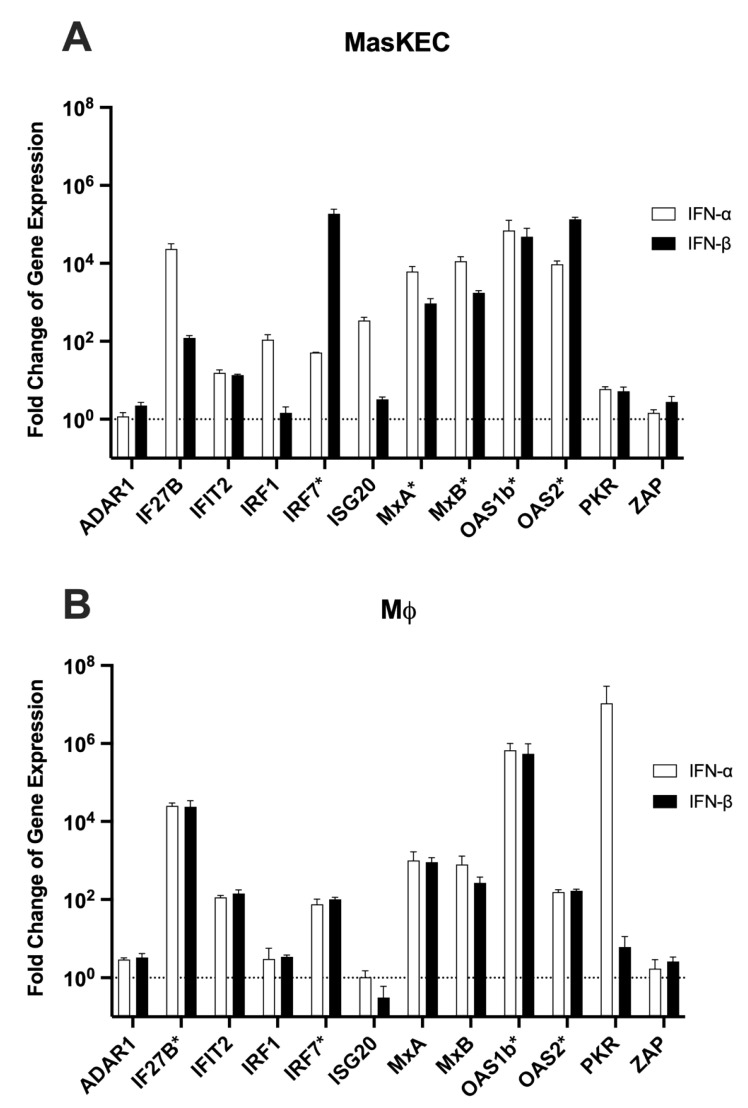
Changes in ISG expression after stimulation with IFN-α or IFN-β. MasKECs (**A**) or MΦs (**B**) were stimulated for 16 h with 500 U/mL IFN-α (clear bars) or IFN-β (black bars). Cells without stimulation were used for later normalisation. Cellular RNA was harvested, depleted of DNA, and used for RT-PCR analysis of ISG expression levels. For the RT-PCR-based assays, 10 ng of overall extracted RNA was used. Normalisation was performed with the ΔΔCt method with the housekeeping gene Ppil4. Genes were marked with an asterisk (*) if no signal Ct value for the controls was obtained due to too low concentrations in unstimulated cells. The previously determined cut-off Ct values (Table 2) were used to calculate the fold change of ISG expression of these genes. The dashed line indicates the ISG level of untreated control cells. The experiment was performed in triplicate. Shown are the mean and standard deviation.

After the multiplex RT-PCR assays were established, different stimulation experiments were performed to verify that the activation of the type I IFN pathway led to an upregulation of the selected ISGs. The stimulation was in a first proof-of-concept experiment performed with murine type I IFNs, which directly bind to the IFN-α/β receptor on the cell surface and activate the production of the ISGs. MasKECs or MΦ were stimulated for 16 h with different amounts of recombinant mouse IFN-α or -β, and the changes in ISG expression were compared to a non-stimulated control (Figure 1 and Figure S1).

The stimulation with type I IFNs increased ISG expression levels up to 10^7^-fold. The overall expression pattern was similar for both tested cell lines, with a trend towards a stronger induction in MasKECs (Tables S2 and S3). In MasKECs, IFN-α stimulation resulted, in most cases, in stronger upregulation of ISG expression. The highest upregulation (up to 1000-fold) could be observed for IFIT2 and IRF1 in both cell lines, whereas changes up to 100-fold was observed for IF27B, MxA, MxB, OAS1b, OAS2, and IRF7. No or only weak upregulation (up to tenfold) was observed for ADAR1, PKR, and ZAP. ISG20 was upregulated after IFN-α treatment in MasKECs. IFN-α treatment in MΦ and IFN-β stimulation in MasKECs, however, resulted in no change in gene expression, and in IFN-β stimulated MΦ, it was downregulated. Stimulation with other IFN concentrations (50, 100, and 1000 U/mL) led to similar results (Figure S1), and only for some genes such as IFIT2, MxA, and MxB, was a concentration-dependent increase seen. As similar ISG expression patterns were observed for MasKECs and MΦ, the following experiments were only performed in MΦ since these cells are thought to be early target cells during LASV infection and are, as antigen-presenting cells, of major relevance for both the early innate immune response and for the priming of T- and B-cells [45,46].

**Figure 2:**
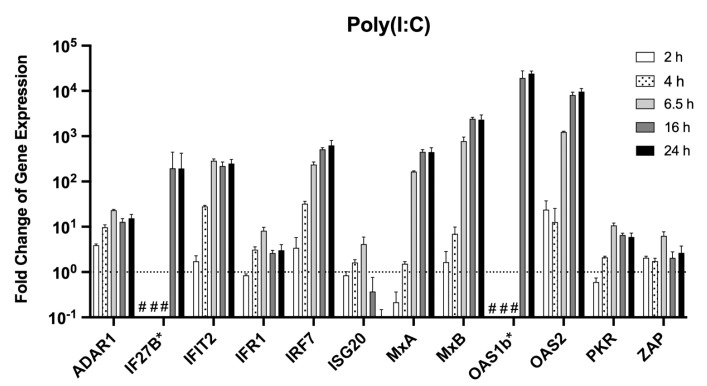
Changes in ISG expression after stimulation of MΦs with Poly(I:C). MΦs were stimulated with 1 µg/mL Poly(I:C) or PBS for the untreated controls for 2, 4, 6.5, 16, or 24 h. Cellular RNA was isolated and depleted of DNA. For the RT-PCR-based assays, 10 ng of overall extracted RNA were used. Normalisation was performed with the ΔΔCt method with the housekeeping gene Ppil4. Genes were marked with an asterisk (*) if no signal Ct value for the controls was obtained due to low concentrations in unstimulated cells. The previously determined cut-off Ct values (Table 2) were used to calculate the fold change of ISG expression of these genes. (#) no Ct values were obtained for the experimental or control-treated cells, and a calculation of the fold change of expression was not feasible. The dashed line indicates the ISG level of untreated control cells. The experiment was performed in triplicate. Shown are the mean and standard deviation.

After confirming that direct treatment with recombinant type I IFN leads to a detectable change in the ISG expression, MΦ were stimulated with Poly(I:C). This double-stranded RNA analogue is known to induce the RIG-I/MDA-5 pathway leading to the production of type I IFN (Figure 2).

ISG expression was weakly induced in Poly(I:C) stimulated MΦ compared to type I IFN stimulated cells. For most genes, an increase over time could be observed, with stable levels mostly reached after 16 h. Especially, IF27B and OAS1b could not be detected earlier than 16 h post-stimulation. Little or no stimulation at all time points was observed for ADAR1, IRF1, ISG20, PKR, and ZAP, whereas strong induction was seen for IFIT2, IRF7, and OAS2 as well as for MxA, MxB, and OAS1b at later time points, which is similar to the results from the IFN-α or -β stimulation.

### 3.3. In Vitro Stimulation with Viruses That Induce an IFN Response

**Figure 3:**
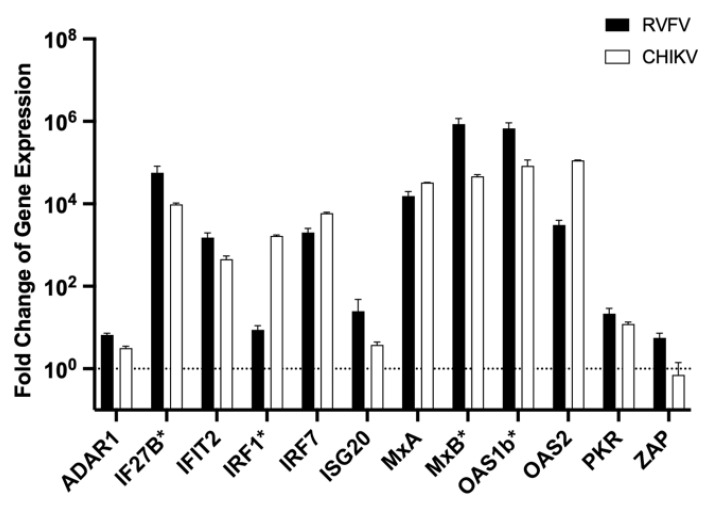
Changes in MΦ ISG expression after infection with RVFV or CHIKV. MΦs were infected with either RVFV or CHIKV with an MOI of 1 and incubated for 24 h. RNA was isolated and depleted of DNA. For the RT-PCR-based assays, 10 ng of overall extracted RNA was used. Normalisation was performed with the ΔΔCt method with the housekeeping gene Ppil4. Genes were marked with an asterisk (*) if no signal Ct value for the controls was obtained due to too low concentrations in uninfected cells. The previously determined cut-off Ct values (Table 2) were used to calculate the fold change of ISG expression of these genes. The dashed line indicates the normal ISG level of untreated control cells. The experiment was performed in triplicate. Shown are the mean and standard deviation.

After the induction of ISG expression was successfully verified for direct receptor activation (addition of IFN-α or -β) or activation of PRRs (Poly(I:C)) stimulation, we wanted to test if ISGs were also expressed in the context of a virus infection. MΦ were infected with an RVFV strain that is deficient in blocking the IFN response due to a mutation in its NS protein [39] or CHIKV, which is known to induce a strong IFN response in human macrophages [47]. Strong upregulation was observed for both viruses 48 h post-infection, with changes in expression levels comparable to those induced by direct stimulation with IFN-α or -β (Figure 3).

Similar to the results from the other stimulation experiment, high expression levels were observed for IF27B, IFIT2, IRF1, IRF7, MxA, MxB, OAS1b, as well as OAS2 and low increases for ADAR1, ISG20, PKR, and ZAP (see Tables S3 and S4 for direct comparison).

### 3.4. Analysis of Arenavirus-Induced Type I IFN Response in M. natalensis Cells

**Figure 4:**
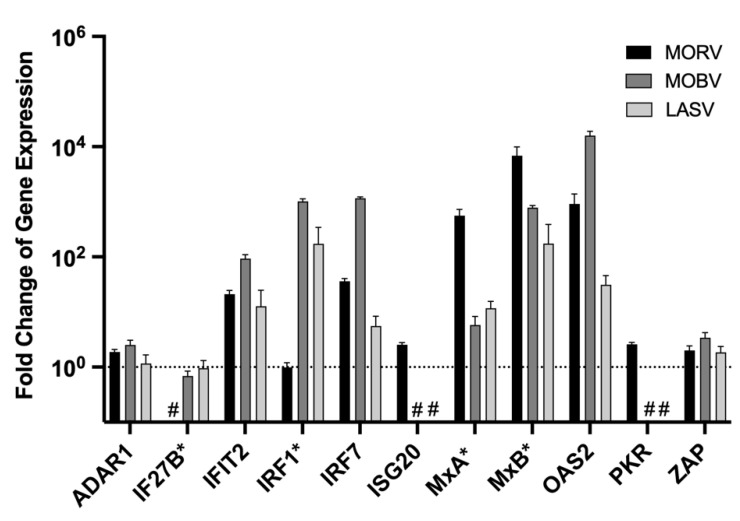
Changes in ISG expression after infection with different arenaviruses. MΦ were infected with either MORV, MOBV, or LASV with an MOI of 1 and incubated for 24 h. RNA was isolated and depleted of DNA. For the RT-PCR-based assays, 10 ng of overall extracted RNA was used. Normalisation was performed with the ΔΔCt method with the housekeeping gene Ppil4. Genes were marked with an asterisk (*) if no signal Ct value for the controls was obtained due to too low concentrations in uninfected cells. The previously determined cut-off Ct values (Table 2) were used to calculate the fold change of ISG expression of these genes. (#) no Ct values were obtained for the experimental and control-treated cells, and a calculation of the fold change of expression was not feasible. The dashed line indicates the normal ISG level of untreated control cells. The experiment was performed in triplicate. Shown are the mean and standard deviation.

To analyse if different arenaviruses have distinct potentials to induce a type I IFN response in *M. natalensis* cells, MΦ were infected with the non-pathogenic, homologous MORV and the heterologous MOBV and the highly pathogenic LASV. For both LASV and MORV, *M. natalensis* is the natural rodent reservoir, while MOBV has been isolated from *Praomys* sp. Changes in ISG expression were analysed 24 and 48 h post-infection (Figure 4 and Figure S2). Virus replication was quantified through RT-PCR, and high amounts of viral RNA (Ct 15–23) were present for all viruses at both time points (Figure S3).

Infection with all arenaviruses induced only a weak to moderate type I IFN response compared to the response observed after RVFV or CHIKV infections, indicating an efficient suppression of the innate immune response. MORV or MOBV infection led to overall higher ISG expression levels compared to the infection with LASV. No differences in the response after 24 (Figure 4) and 48 h (Figure S2) were observed. In contrast to the previous stimulation or infection experiment, OAS1b could not be detected in any of the arenavirus-infected samples (not shown in the figure).

**Figure 5:**
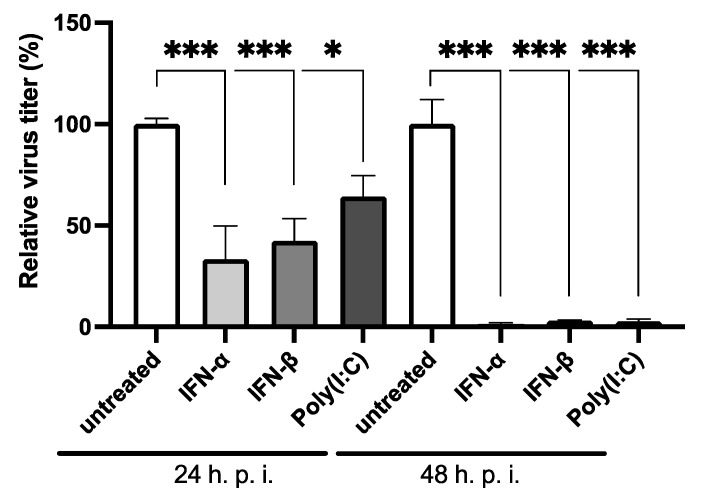
Infection of stimulated MΦ with LASV. MΦ were stimulated with 500 U/mL IFN-α, or IFN-β or Poly(I:C) and sixteen hours post-stimulation, they were infected with LASV with an MOI of 0.1. The cell culture supernatant was collected 24 or 48 h post-infection (h. p. i.) and used for immunofocus assay. The virus titres are shown relative to their uninfected controls. The experiment was performed in triplicate. Shown are the mean and standard deviation. Differences in virus titres were analysed with one-way ANOVA and Bonferroni’s multiple comparison test. *, *p* < 0.033; ***, *p* < 0.001.

To verify that LASV replication can be efficiently controlled by the type I IFN response in *M. natalensis* cells and thus depends on the suppression of this antiviral defence mechanism, we stimulated MΦ with IFN-α, IFN-β, or Poly(I:C) and infected them subsequently with LASV. Virus replication was assessed 24 and 48 h post-infection (Figure 5).

LASV replication was significantly reduced in stimulated MΦs, which was more pronounced 48 h post-infection. The capacity of IFN-α or -β to suppress LASV replication was more substantial than that of Poly(I:C) early after infection but similar at the later time point.

## 4. Discussion

The rising number of spillovers of zoonotic viruses into the human population emphasises that a better understanding of factors restricting host switching is urgently needed. *M. natalensis* is the natural host for several pathogenic and non-pathogenic arenaviruses such as LASV, MOPV, or MORV [11,16]. However, some LASV strains, as well as MOBV, have different rodent hosts [12,13,15], making arenaviruses and *M. natalensis* an ideal model to study the underlying mechanism behind virus–host barriers. As for many other natural host species, such as bats, tools and reagents to study immunology in these animals are scarce. Our aim was to develop and validate RT-PCR assays to detect changes in ISG expression in *M. natalensis* cells as a tool to investigate the role of the type I IFN response for virus–host restrictions [48].

Based on transcriptomic analysis, we designed primers and probes that detect 12 relevant ISGs. Both the direct activation of the IFN-α/β receptor through the exogenous addition of type I IFNs and the activation of RIG-I/MDA5 with Poly(I:C) resulted in an upregulation of the ISG expression in *M. natalensis* cells. MΦ and MasKECs were sensitive to IFN treatment, and the lowest tested concentration already led to strong ISG expression with little dose dependency for most genes. The expression levels in MasKECs and MΦ after IFN-α treatment were similar to what has been described for murine fibroblasts, which showed weak upregulation of IFIT2, moderate upregulation of OAS1b and 2 and strong upregulation of IRF7 and Mx2 (equivalent to MxB in rodents) [49]. We also could activate the IFN system through the infection with CHIKV and an RVFV strain, which is deficient in blocking the IFN system. Both viruses induce a strong IFN response in *M. natalensis* MΦ with an above 1000-fold upregulation of IFIT2, OAS2, OAS1b, and MxB. The infection of human HEK-239T cells with RVFV in contrast, led to a below 10-fold upregulation of these genes [50]. The infection of human macrophages with CHIKV resulted in a below 100-fold increase in OAS1 compared to the above 80,000-fold increase in *Mastomys* cells [47], showing the responsiveness of these cells to virus-induced innate immune responses.

To investigate the role of the IFN system for host switching and virus–host restrictions for arenaviruses, we used the homologous LASV and MORV as well as the heterologous MOBV, the last two being non-pathogenic, as model viruses. In our infection experiments in *M. natalensis* cells, we observed an overall weaker ISG upregulation after arenavirus infection compared to RVFV or CHIKV infection and IFN-α/β or Poly(I:C) treatment. ISG expression levels were similar after infection with homologous and heterologous viruses, and the response induced by MOBV closely resembled that of MORV infected cells. The IFN response induced by arenavirus infection of *M. natalensis* cells was, in our experiments, not dependent on the rodent host of the virus. The activation of the type I IFN system, however, was stronger after infection with the non-pathogenic viruses MORV and MOBV than after infection with LASV. This is in accordance with in vitro data from infected human cells. Macrophages and endothelial cells are activated by MOPV infection, another apathogenic arenavirus, but not by LASV infection. MOPV but not LASV infection also leads to the activation of plasmacytoid dendritic cells, which produce large quantities of pro-inflammatory cytokines, especially type I IFN, upon activation and thus drive innate immune responses [33,45,51,52,53]. In vivo experiments with non-human primates and mice also show that the efficient suppression of the IFN response is a key factor for pathogenicity for LASV and essential for efficient virus replication [54,55,56]. In line with these findings was the suppression of the IFN system required for LASV replication in *Mastomys* MΦ, and activation of the IFN response before infection significantly reduced virus titres. A comparable reduction in virus titres was observed for activation at the interferon receptor (treatment with IFN-α/-β) and the PRR level (treatment with Poly(I:C)).

Efficient suppression of the IFN response to overcome the sensitivity to IFN-α or -β induced antiviral responses is a common feature of many highly pathogenic RNA viruses such as hantaviruses, SARS-CoV-2, Nipah, Ebola and Marburg virus or Flaviviruses such as Dengue or Zika virus [57,58,59,60,61,62,63]. SARS-CoV-2 completely inhibits the expression of IFN-I and ISGs in infected human epithelial airway cells [57]. Plasmacytoid dendritic cells (pDC), in contrast, are refractory to infection but can sense SARS-CoV-2 and produce IFN-α as well as other pro-inflammatory cytokines that protect surrounding epithelial cells from infection [64,65]. Similar to the data from our infection experiments were IFIT2 and MxB upregulated in these pDCs. Filoviruses, in contrast, completely block ISG expression in infected DCs, even after exogenous activation of RIG-I [66].

In compliance with in vitro data in human cells and data from in vivo experiments, we observed that ISG upregulation in infected *M. natalensis* MΦ aligns with the pathology of the tested arenaviruses. The two non-pathogenic viruses induced stronger responses than the pathogenic LASV. In this regard, Old World arenaviruses behave similarly to other bunyaviruses, such as hantaviruses, where the ability to regulate early IFN responses has also been identified as one of the major virulence factors. Infection of endothelial cells with non-pathogenic hantaviruses such as Prospect Hill virus (PHV) leads to a stronger and more diversified ISG expression compared to the infection with pathogenic viruses such as Andes virus (ANDV). Especially, transcripts for MxA and B were >100-fold more abundant one day post-infection with PHV compared to an increase of <2-fold for ANDV [67].

In contrast to what was observed in infection experiments in *Mastomys*, where the homologous MORV but not the heterologous MOBV was able to establish persistent infection, we did not detect relevant differences in vitro in ISG expression between the two viruses [40]. In vitro, homologous and heterologous viruses induced IFN responses to a similar degree. This suggests that the type I IFN system plays only a minor role in restricting arenaviruses to their natural host. However, all experiments were performed with isolated cells and not in the context of an entire animal, and we only looked at ISG transcription. Infection experiments with *M. natalensis* with homologous and heterologous arenaviruses will be needed to fully understand the role of the IFN system in Arenavirus–host restrictions. It is also possible that differences at the protein level differ from the observed changes at the transcript level. For Zika virus, it is described that IFN-I protein concentrations remain unchanged despite their transcriptional upregulation [59]. Cytokines that would induce an adaptive immune response also remain to be investigated. To analyse the adaptive and humoral immune responses as further candidates restricting arenaviruses from switching hosts, in vivo experiments will be needed to capture the interaction of different cell types and organs. The development of qRT-PCR assays based on homology sequences from *M. coucha*, mouse and rat described in this study should be easily adapted to further analyse cytokines and chemokines. Together with the already established and validated RT-PCR assays, they will be a valuable tool to further the investigation of the role of the *M. natalensis* immune response in virus–host barriers.

## Figures and Tables

**Table 1:** Cycling conditions for the ISG RT-PCR-based assays.

Step	Temperature	Time
Reverse transcription	50 °C	15 min
Activation	95 °C	2 min
Cycling (45×)	95 °C	15 s
	60 °C	30 s

**Table 2:** Primer efficiencies and limits of detection of the RT-PCR-based assays.

Gene Targeted	Primer Efficiency	Ct value of Detection Limit (50% Detection)
60S ^1^	2.3	40.4
Ppil4 ^1^	2.0	41.3
ADAR1	2.0	39.1
IFIT2	2.1	35.2
IF27B	2.3	42.1
IRF1	2.0	43.1
IRF7 ^2^	1.8	43.1
ISG20	3.0	40.4
MxA ^2^	1.8	42.1
MxB ^2^	2.0	40.4
OAS1b	1.9	42.7
OAS2	1.9	41.9
PKR ^2^	1.7	38.5
ZAP	1.8	36.7

^1^ Housekeeping gene. ^2^ RNA from stimulated cells was used to determine primer efficiency.

## Data Availability

Sequences of *M. natalensis* ISGs and housekeeping genes with indications for primer and probe binding sites are available as Appendix A.

## Supplementary Materials

The following supporting information can be downloaded at: https://www.mdpi.com/article/10.3390/v14091986/s1, Table S1: Master mix set-up for the RT-PCR. Table S2: Changes in ISG expression of MasKECs after stimulation with IFN-α or IFN-β. Table S3: Changes in ISG expression of MΦ after stimulation with IFN-α, IFN-β or Poly(I:C). Table S4: Changes in ISG expression of MF after infection with MORV, MOBV, LASV, RVFV or CHIKV. Figure S1: Changes in ISG expression after stimulation with IFN-α or IFN-β. Figure S2: Changes in ISG expression after infection with different arenaviruses. Figure S3: Confirmation of efficient infection with arenaviruses.

## Appendix A

mRNA sequences of the genes analysed in this study. The primer and probe binding regions are marked.

**Fwd primer**//Rev primer//Probe

***60S Ribosomal Protein L13a***

ATGGCGGAGGGGCAGGTTCTGATACTGGATGGCCGAGGCCATCTCCTGGGCCGCTTGGCGGCCATTGTGGCCAAGCAGGTACTGCTGGGCCGGAAGGTGGTGGTTGTACGCTGTGAGGGCATCAACATTTCTGGAAATTTCTACAGAAACAAGTTAAAGTATCTGGCCTTTCTTCGAAAGCG**GATGAATACCAACCCCTCTCG**AGG**ACCCTATCACTTCCGAGCCCC**CAGCCGCATTTTTTGGCGCACTGTGCGGGGCATGCTGCCCCACAAGACCAAGAGAGGCCAGGCTGCCCTGGAACGC**CTCAAGGTGTTGGATGGGAT**CCCTCCACCCTATGACAAGAAAAAGCGGATGGTGGTTCCTGCTGCCCTCAAGGTCGTTCGACTGAAGCCTACCAGGAAGTTTGCTTACCTGGGGCGTCTGGCTCATGAGGTTGGGTGGAAGTACCAGGCAGTGACAGCCACTCTGGAGGAGAAACGGAAGGAAAAGGCCAAGATCCATTATCGGAAGAAGAAGCAGCTCTTGAGGCTACGGAAACAGGCAGAAAAGAATGTGGAGAAGAAAATCTGCAAGTTCACAGAGGTCCTCAAGACCAATGGACTCCTGGTGTGA

***Peptidylprolyl Isomerase Like 4 (Ppil4)***

GCCGGCGCCATGGCGGTTCTCTTGGAGACCACTCTGGGAGACGTGGTCATCGATTTGTACACCGAAGAGCGTCCACGTGCTTGCTTGAATTTTTTGAAGCTGTGCAAAATAAAATATTACAACTATTGCCTCATACACAATGTACAGAGGGACTTTATCATACAAACAGGAGATCCCACAGGGACTGGCCGTGGGGGAGAGTCTGTATTTGGACAACTGTATGGTGATCAAGCAAGCTTTTTTGAGGCAGAAAAGGTGCCAAGGATAAAGCACAAGAAGAAAGGCACTGTGTCCATGGTGAACAATGGCAGTGATCAGCATGGATCTCAGTTTCTTATTACTACAGGAGAAAATTTAGATTACCTTGATGGTGTTCATACAGTGTTTGGCGAAGTGACAGAAGGCATGGACATAGTTAAGAAAATCAATGAGACCTTTGTGGACAAGGATTTTGTACCATATCAAGATATCAGGATAAATCATACAGTGATTTTAGATGATCCCTTTGATGACCCTCCTGATTTACTAATTCCTGATCGATCACCAGAACCTACAAAGGAACAATTAGATAGTGGTCGAATAGGAGCAGATGAAGAAATTGATGACTTCAAAGGAAGATCAGCTGAAGAAGTAGAAGAAATAAAGGCAGAAAAAGAGGCTAAAACTCAAGCTATTCTCTTAGAGATG**GTGGGAGACTTACCTGATGC**AGATATTAA**GCCTCCAGAAAATGTGCTGTTTGTGT**GTAAATT**GAATCCAGTGACCACAGATGAG**GACTTGGAGATAATATTCTCAAGATTTGGGCCAATAAGAAGGTAA

***Adenosine Deaminase (ADAR1)***

ATGCTACTCCTTTCCAGGTCCCCAGATGCACATCCAAAGACACTTCCTCTCACTGGCAGCACCTTCCATGACCAGATGGCCATGCTGAGCCACAGGTGCTTCAATGCCCTGACCAACAGTTTCCAGCCCTCCCTGCTCGGCCGCAAGATCCTGGCTGCCATCATTATGAAGAGAAATGCTGAGGACATGGGTGTTGTCGTGAGCTTGGGGACAGGGAATCGCTGTGTGAAAGGGGACTCTCTCAGCCTAAAGGGAGAGACAGTCAATGACTGCCATGCCGAAATCATCTCCCGGAGGGGCTTCATCAGGTTTCTCTACAGTGAACTGATGAAGTACAACCACCACACTGCCAAGAACAGCATATTTGAGCTGGCCAGGGGAGGAGAGAAGCTGCAGATAAAAAAGACGGTTTCTTTTCATCTCTACATCAGCACGGCGCCATGTGGAGATGGAGCCCTCTTTGACAAATCCTGCAGTGACCGAGCTGTGGAAAGCACAGAGTCCCGCCATTACCCTGTTTTTGAAAATCCCAAACAAGGCAAGCTTCGCACCAAGGTGGAGAATGGGGAAGGCACAATTCCTGTGGAGTCCAGTGACATTGTACCCACGTGGGATGGCATCCGGCTTGGGGAGAGACTCCGTACCATGTCCTGTAGTGACAAAATCCTACGCTGGAATGTGCTGGGCCTGCAAGGGGCGCTGTTGACACACTTCCTACAGCCTGTGTACCTGAAATCTGTAACATTAGGTTACCTTTTCAGCCAAGGGCATCTGACCCGAGCTATTTGCTGCCGTGTGACCAGAGATGGGAAAGCATTTGAGGATGGACTACGCTATCCCTTTATTGTCAACCACCCCAAGGTCGGCCGAGTCAGTGTGTATGATTCCAAAAGGCAGTCCGGAAAGACCAAGGAGACAAGCGTCAACTGGTGCTTGGCCGATGGCTATGACCTAGAGATCCTGGATGGCACCAGAGGCACTGTGGATGGACCCGGGAAAGAGTTGTCTCGGGTGTCCAAGAAGAATATTTTCCTTCAGTTTAAGAAGCTCTGCTCCTTCCGAGCCCGCAGAGATTTGCTGCAGCTCTCCTATGGTGAGGCCAAGAAAG**CTGCCCGTGACTATGACTTAG**CCAAGAACT**ACTTCAAGAAAAGCCTTCGGGATATGGGCTATGGGAACTGGATCAGCAA**ACCCCAGGAGGAAAAGAACTTTTATCTCTGTCCAGTACCCAATGACTGA

***Interferon Alpha-Inducible Protein 27B (IF27B)***

ATGTCCAAAATTCAAATGATGCCTCTTACAACCTCGAGTTTCCTGCCCTCTGGAGGCTCCTGCAGCTCCTCTAGCTCCTGCAGCTCCTCTAGCTCTTGGGGTTCTGGTTCCTGCTCTCCAGAGGCTCCTGTGGCTCCTGCAGCTCCTGGGGCTCCTGCTGCTTCTGGGTCTCCTGCGGCTCCTGTGGCTTCTGCAGCCCTTGCGCCTCCTGCAGCTCCTGCAGCTCCTGAGGCTCCTGCAGCTTCTGGGTCTCCTGCGGCTCCTGCAGCTTCTGCAGCTCCTGCAACTGTAGCTCCTGCAGCTGCTCCAACAGCCCCCAGGATGATG**TTGGTTGCTGTGGAGAGTC**CAAGGACCCCCACTGACTGGAGTGTGGCTACCAGGCTTCCTGCTGCAACTCCGCCCCCATTGGCAATTGCTGCAGC**AGACATCATCTTGGCTGCTATGGATGC**AGCTG**CAATGCCTGTTCCAGTGAAG**CCTATGGCAGTCAGGGTGACTGGTACAGCTGCCACAGCCAGGGCTATAGGGAGTAGAGGTAGGTGA

***Interferon Induced Protein with Tetratricopeptide Repeats 2 (IFIT2)***

ATGAGTACCACCACGAAGGAGTCATTGGAGAGCAGTCTACGGCAGCTAAAATGCCATTTCACCTGGAACCTGATAGCAGAAGATGAATCCTTGGATGAGTTTGAGGACAGGGTGTTTAACAAGGATGAGTTTCAGAACAGTGAGTTTAAAGCCACCATGTGCAACATATTGGCCTATGTAAAGCATCGCAGAGGTCTGAATGAGGTGGCGCTGAAATGCCTAGGGGAAGCCGAAGACTTCATCCGACAACAGCATCCTGACCAAGTAGAAATCAAAAGTCTGGTCACTTGGGGAAACTACGCTTGGGTCTACTACCACATGGGCCAGCTCTCAAAAGCTCAGGCTTATCTGGATAAGGTGAAAGAGGTCTGCAAGAAGTTTTCCAGTCCCTACAGGATCGAGAATCCTGCGCTTGACTGTGAGGAAGGGTGGGCCCGATTAAAGTGTACCAAGAACCAAAACGAGAGAGTGAAGGTGTGCTTCCAGAAGGCTCTGGAAAAGGACCCGAAGAACCCAGAATTCACCTCTGGATGGGCCATTGCGAACTATCGTCTGGATGACTGGCCAGCAAGGAATGACTATATTGACTCTCTGAAGCAAGCCATTAGTCTGTCTCCTGACAACACTTACATTAAAGTCCTCTTGGCACTGAAGCTTGATGGGATGTATGAAAAACAAGCAAAGGAGCTAGTTGAAGAAGCCTTAAAGAAAGACCCAAGTGCAATAGATACACTGCTCAGTGCGGCCAGGTTTTATTACAAGATACATGACACAGACAGAGCCATACAGTTGCTTAGAAAGGCTTTGGAATCCCTGCCAGACAATGCTTATGTGCATTACTATATTGGATGCTGTTACAGGGCAAAATTCTTTCAAATAGACACAAGGGAAAGGGCATTTAATGGAGATAGGAAGAAGATAGAGGAACTAATGCAACTAGCACTGGATCACTTACGGAAAGCAGAGGAAATCAAGGAGATGATCGAAAATTCCTGTAGCTTCCTTGCTGGTCTTTATGTTATAAAAGAGCAGTATGAAGAAGCCGATTATTACTTCCAGAAAGAATTCAACAAGGACCTCC**CTCCTGGCCCTAAACAGTTACTCCACCTTCGGTATGGCAACTTTCAG**TTTTTT**CAAATGAAGCGTGAAGACAAGG**CTGTCTACCATTATATGGAGGGTGTGAAAATAAAGAGGAAAACAAAGCCAAGAGAAAAGATGAGAGAGAAACTCCAAAGAATCGCCCGAAGGCGGCTTTATAAAGATGAGTCTGATTCTGAAGCCTTGAGCATCTTGGCATTTCTTCAGGAGAACAGAGAAGGTCAGCAAGCTGATGAAGACGTGGAGTCTGTAAACCAGGTCCTTTCAGCATCTCTGGATGAAGCAGGTGCTGAATACAAGTGA

***Interferon Regulatory Factor 1 (IRF1)***

ATGCATTTACTCTTCCGTTCTATAGGCCGATACAAAGCAGGGGAAAAAGAGCCAGATCCCAAGACATGGAAGG**CAAACTTCCGTTGTGCCATG**AACTCCCT**ACCGGACATCGAGGAAGTGAAGGA**C**CAGAGCAAGAACAAGGGCA**GCTCTGCTGTACGGGTGTACCGGATGCTGCCACCCCTCACGAAGAACCAGAGGAAAGGTAGCCAAGGGCACTGGGTCCTCGGGAGGAAGGGAAGGGAAGAAGGGGCAGAGAGAAGATCAGCTGCCGCTCACTGCTTGTTCAACCCGCAGAGAGAAAGTCCAAGTCCAGCCGAGACACTAAGAGCAAAGCCAAGAGGAAGGTGA

***Interferon Regulatory Factor (IRF7)***

A**TGGAAAATAGGGAAGAGGTGAC**CCCCAGCAATGGCCCATCCACACAGGGTGTGTCCCTAGGATCATTCCTGGCAAGAGAAAATGCTGGG**CTCCAAACCCCAAGCCCTCTGCTTTCTAGTGATGCTGGGGAC**CTCTTGCTTCAGGTTCTGCAATACAGCCATATACTGGAATCTGAGTCTGGGGCAGACTCCGTCCCCCCACAGGCTCCTGGCCAAGAGCAAGAACATATTTCTGAAGAATCCTATGGAGCATGGCAGGTGGAAGCTGTCCCCAGTCCCAGGCGTCAACACCCAGCTCTGATGACCGAGAGCAACCTTGGGTTCCTGGATGTGACCATCATGTACAAAGGCCGCACAGTGCTACATGCAATGGTGTGGCACCCCAGATGTGTGTTCCTGTATAGCCCCATGGGCTCAGCAATAAAAACTTCAGAGCCCCAGCCGGTGATTTTTCCCAGCCCTGCTGAGCTCCCAGATCAGAAGCAGCTGCACTACACAGAGACTCTTCTACAGCATGTGTCTCCAGGCCTTCAGCTGGAGCTTCGGGGACCGTCACTGTGGGCCCTGCGTATGGGCAAGTGCAAGGTGTACTGGGAGGTGGGCAGCCCGATGGGCACCACCAACCCCTCCACCCCAGCCCAGCTGTTGGAACGCAACTGCCATACCCCCATTTTTGACTTCAGCACTTTCTTCCGAGAACTGGAGGAGTTCCGGGCTCGGAGGCGGCAAGGATCACCACACTACACCATCTACCTGGGTTTTGGGCAAGACTTGTCAGCAGGGAGGCCCAAAGAGAAGAGCCTGATCCTGGTGAAGCTGGAGCCATGGCTATGCAAGGCACACCTGGAAGGCGTGCAGCGTGAGGGTGCGTCCTCCCTGGACAGCAGCAGTCTTGGCCTGTGTTTGTCTAGCACCAATAGTCTCTATGATGACATTGAACACTTCCTCATGGATCTGGATCAGTGGGCTTGA

***Interferon Stimulated Exonuclease Gene 20 (ISG20)***

ATGGCAGGCAGCCCAGAGGTGGTAGCCATGGACTGTGAGATGGTGGGGCTTGGGCCTCAAAGGGTAAGTGGCCTCGCCCGCTGCAGCATCGTCAACTTCCATGGCGCAGTCCTGTATGACAAGTACATTCAGCCTGAGGGAGAGATCACGG**ATTATAGAACCCAAGTCAGCGG**GATCACGCCTC**AGCACATGGTGAGGGCCACA**CCATTTGGTGAAGCCAGGCTAGAGATCCTGCAGCTTCTGACAGGCAAGCTGGTGGTGGGC**CATGACCTGAAGCACGACT**TCAATGCCCTGAAGGAAGATATGAGCAAATACACCATCTATGACACGTCCACAGACATGCTGCTATGGCAGGAAGCCAAACTGCAGTACTACAACCGCGTGTCCCTGAGGCTGCTGTGTAAGCGCCTACTACACAGGAGCATCCAGAACAACTGGCGGGGCCACTGCTCTGTGGAAGATGCCAGGGCCACGATGGAGCTCTACAAAATCTCTCAGCGACTCAGAGCCCAGGGAGGGCTGTCCCGCCTGGGGACATCAGACTGA

***Interferon-Induced GTP-Binding Protein MxA (MxA)***

ATGTCCTTTCTGATAAATAAAATCAATGTCTTCAACAAGGACATCACGACTTTGGTACAAGCACAGGAAACCATATCAGGGGGAAACAGCCGGTTGTTTACCAAACTTCGAGATGAATTTTTTGCTTGGGATGCTTTTATTGAGAAACATTTCAAAGAAAGTTCTCCTGAGATACATAGCAAGATGAAAGAATTTGAAAATCAGTATCGTGGCCGGGAGCTGCCAGGGTTTGTGGACTATAAGGCATTTGAGAACATCATTAAAAAGCAAGTCGAAGCCCTGGAAGAGTCCGCTGTGAATATGCTGCGCAATGTCACTGAGATGGTCCGAACCGCCTTCATAAAGGTGTCATCAAACAATTTTGGTAATTTTTCAAACCTCCACTATACTGCTAAGTCCACAATTGAAGACATAAGATTAAACCAAGAAAAAAAAGCTGAGAAACTGATCCGACTTCACTTCCAGATGGAACAGATTGTCTACTGCCAGGACCAAATTTACAAGGAAGCCTTGAAGACAATCAGAGAGGAGGAGGCAGAGAAAGAGAAGACTAGGAGGCTAAATAACTCTCAGTTTC**CTCAAAAGGAGTGGACTAGCCCAGAGATGAGCCAGCACCTGAAT**GCTTACTACCAGGAGTG**CAGAAGGAACATTGGGAGACA**GATCCCTCTGATCATCCAGTACTTCATCCTGAAAACATTTGGGGAGGAAATGGAGAAAGCCATGCTTCAGCTTTTACAGGACACCCATAAATGCAGCTGGTTCCTGCAGGAGCAGAATGACACCAGAGAGAAGAAGAAGTTCCTGAAGAGGCGGCTTTTAAGGCTGGATGAGGCTCGGCGGAAGCTTGCCAAATTCTCCGATTAA

***Interferon-Induced GTP-Binding Protein MxB (MxB)***

ATGGATTCTGTGAACAATCTCTGCAGTCAGTATGAAGAGAAAGTGCGGCCCTGCATTGACCTCATTGACTCCCTGCGGGCTCTGGGTGTGGAGCAGGACCTGGCCTTACCTGCCATCGCTGTCATTGGGGACCAGAGTTCAGGGAAGAGCTCTGTGCTGGAAGCACTGTCTGGAGTGGCCCTCCCTAGAGGCAGTGGTATCGTTACCCGGTGCCCTCTGGTGCTGAAACTGAAGCAACTCAAGAAGGCAGCAGAATGGAGAGGCAAGGTCATCTATAAGGACACCGAGATTGTGATCTCACACCC**CTCACTGGTAGAAAGGGAAGTC**AAT**AAAGCCCAGAACTTGATTGCCGG**GGAAGGGTTGCAGATTAGCTCTGAGCTCATTAGCTTGGAGATCAGCTCTCCACATGTCCCA**GACCTGACTCTGATTGACCTG**CCTGGTATCACAAGGGTGGCTGTAGGTGACCAGCCAGCAGACATTGAATACAAGATCAAGAGACTCATCAGGACATACATCCAAAAACAGGAGACCATCAACCTGGTGGTTGTCCCCAGCAATGTGGACATTGCCACCACAGAGGCCCTGAGCATGGCTCAGGAGGTGGACCCTGAAGGGGATAGGACCATAGGGATCTTGACCAAGCCTGATCTGGTGGACAGAGGTGCTGAAGACAAGGTTGTTGATGTGGTGCGGAACCTGGTGTGCCATCTGAAAAAGGGCTACATGATAGTCAAGTGCAGGGGTCAGCAGGACATCCAGGAGCAGTTGAGCTTGGCTGAGGCTCTTCAGAATGAGCAAGTCTTCTTCAAGGAGCACCCTCATTTCAGAGTTCTTCTGGAGGATGGGAAGGCTACAGTGCCCTGCCTGGCAGAGAGACTGACCACGGAACTCATCGCGCACATCTGTAAATCTCTTCCTTTGTTAGAAAATCAAATAAAGGAAAGTCACCAGAGTGCAAGTGAAGAGCTACAGAAGTACGGTATGGACATACCAGAGGATGACAGCGAAAAAACCTTCTTCCTCATCGAAAAAATCAATGCTTTTAACCAGGATATCAAAGCCATAGTAGAAGGGGAAGAGCATGTGGGGGAGGGAGAATGTCGCCTATTCACCAGGCTCCGAAAAGAGTTCTTGTCCTGGAGTATGGAGATTGAAAAGAATTTCCAAAAAGGTTATGATGTTTTATATAAAGAAGTCTGGATGTTTGAAAGGCAGTACCGAGGCCGAGAACTGCCAGGGTTTGTGAATTACAAAACATTTGAGAACATCATAAGAAAACAAATCAAAATCCTGGAAGATCCAGCTATAGAAATGCTGCACAGAGTCACTGAAATTGTACGAGTTGCCTTTACACGTGTTTCTGAGAAAAATTTTTGTGAGTTTTTTAACCTCCACAGAACTACCAAGTCCAAACTTGAAGACATCAGATTAGAACAAGAGAAGGAAGCAGAGAAGTCGATCCGACTTCACTTCCAGATGGAACAAATCATCTACTGCCAGGACCAAATTTACAGAGGATCCCTGAAGAAGGTCAGAGAGGAAGAAGCTGAGGAGGAGGAGAAGAAGAAGAAGCATGACCTCGTCAGTTCCTCTGAGTCTGAAGATTTACAGAACTCATCCATGGCCGAGATCTTCCAGCATCTGAATGCCTACCGCCAGGAGGCCCACAACCGCATCTCCAGCTACATTCCCTTGATCATCCAGTATTTCATGCTGAAGACGTTTGCTGATCAGCTGCAGAAGGACATGCTCCAGCTCCTGCAGGACAAGGATTCCTGCAGCTGGCTCCTGAAGGAGCAGAGTGACACCAGCGAGAAGAGGAAATTCCTGAAGGAGCGGCTGGCAAGGCTGGCCCAAGCTCGGCGCCGGCTAGCAAAATTCCCTGGTTAA

***2′-5′-Oligoadenylate Synthetase 1b (OAS1b)***

TCTGGAACTATCATGGAGCAGAGACTCAGGAGCATCCCGGCCTCCGAGCTCGATAAGTTCATAGAGAATCATCTTCCGGACACCAGCTTCTGTGCTGACCTCAGAGAAGTCATAGATGTCCTATGTGCTCTCCTGAAAGACAAATCCTTCCGGAGCTTTCCCGGCCCAGTGAGGGTCTCCAAGGTGGTGAAGGGTGGCTCCTCAGGCAAAGGCACAGCGCTCAAGGGCAGGTCACACGCTGACCTGGTGGTGTTCCTTGACAATCTCACCTACTTTGAGGATCGGTTAAACAAACAGGGAGAGTTGATTAAGGAAATTAAGAAACAGCTGTATGAGATTCAGCATGACAGACATTTTG**GAGTGAAGTTTGAGGTCCAGAG**TCCATGGTGGCCCAACCCCCGGGCTCTGAGCTTCAAGCTCAGCGCCCCCCACCTTCATCAGGAGGTGGAGTTTGA**TGTGCTTCCAGCCTTTGATGTCCT**G**GGACAGGTTAGCATCTACAGC**AAGCCTGACCCCCAAATATATGCCCTCCTCATCAGGGAATGTGTCTCCCTGGGGCTGGAGGGCGAGTTCTCTGCCTGCTTCACAGAGCTCCAGCGGAACTTCCTGAAGCAGCGCCCAACCAAGCTGAAGAGTCTAATACGCCTGGTCAAGCACTGGTACCAACTGTGTAAGGAGAAGCTGGGGAAGCCACTGCCCCCACAGTATGCCCTGGAGCTGCTCACAATCTATGCCTGGGAGTGTGGGGGTCGAATAACTAAATTCAACACAGCCCAGGGCTTCCGGACTGTCTTGGAACTGATCACCAAATACAAACAGCTTCAAATCTACTGGATAGTGTGCTATGACTTCCTACACCCAGAGGTCTCCGATTACCTGCTCTTACAGCTCAGAAAAGCCAGGCCTGTGATCCTGGACCCGGCTGACCCAACATGGAATGTGGCGGGTTTGAACACAAAGGGCTGGCGGCGACTAGCAGGAGAGGCTGCCGCCTGGCTGCAGTACCCATGCTTTAAGTACAGGGACCGTTCCCGGGTGTGCTCCTGGGATGTGCCGATGGAGGTTGGCGTGCCAAAGCAGAAGAATCTCTTTTATCGTATTTTCTGGATATTGTTTTGGTTTTTGTTTCAGTTCATTTTTGGGAAGACTTCATCTGTGTAG

***2′-5′-Oligoadenylate Synthetase 2 (OAS2)***

ATGGGAAACTGGCTGACTGGCTGGTCATCTGAAGGGCCATCTGGCTATTCATCTGGCTGGTCATCTGGTGGATCTTCAGGGGTGCCCTCCGGGCCAGCACACAAGCTAGAAGAGTCTATCCAGGCAAACCGCCCACCCAGCGAAAAATGTCTGAAGCAGACTGGGGTGTTCTCGGTGACACCCCAGAAGCTAGAAGAGTATATCCAGGCATATCTCAAACCCAATGAAGAATCTCTGAAGCAGCTAGACCAGGCCGTGGATGCCATCTCTGAACTGCTGCTTCGCAGTGAGATCCCTGTGATGAAAGTGGCTAAGGGTGGCTCCTATGGCCGGGAAACAGTCCTAAGAGGCTGCTCCGATGGTACCCTTGTTCTCTTCATGGATTGCTTCCAACAGTTTCAGGATCAGAAGAAATACCAAGTTGAACTCCTTGACACCATTGAACAATGGCTGAAAAGCTATGAGAAATACAAGAAGTCAGTAAACCGTGGACGTGCCCTTGTAGTACAAGTGTCTGTACCAGGGCAGAGTATATTCTTGCAGTTACTGCCAGCCTTCAATCCTCTGCGCTTCAGTGAGAATCCAAGCTCCTGTGTCTATCGGGATCTCAAGCAATCCATGGATCGAGTAGGAGCATCACCAGGGGAGTTCTCAGGCTGCTTCACCACACTGCAGCAGCAGTTTTTCAAGAAATATCCCAGAAGACTGAAGGATTTGATCCTATTGGTCAAGCACTGGTATGAACAGTACCAGGAGAAGCTCCCACCTCAGCCATTGCTATACGCCCTGGAGCTGCTCACTGTGTACGCCTGGGAACAGGGCTGCCAAGCTGAAGACTTCAACATGGCACAAGGCATCAGGGCCGTGCTACAACTCATCAGTCAGCCGACAAATCTGTGTGTCTACTGGACCGTCAATTACAACTTTGAGGATAAGACAGTCCAGAACATCCTTCTGTACCAGCTAAACTCTCCAAGACCGGTCATCTTGGATCCAACTGACCCAACCAACAATGTGGGAAAAGATGTTAGGCTACTGACAGAAGAGGCTCTGACCTGGCTAAACTCTTCCAGCCTGAATAATGAGTTACCTGCACCATATTGGGATGTTCTGCCCGTACCATTTTTCAATACTCCAAGCCATTTACTGGACAAGTTCATCGATGACTTCCTCCAGCCCAACAAGGCCTTTCTAGGTCAGATCAAAAAAGCCGTTGACATGATCTGCTCCTTCCTTAAAAATAACTGCTTCCGGTATTCTGACACCAAAGTCCTGAAGACCATCAAGGGAGGATCCACTGCCAAAGGCACAGCTCTGAAGCAGGGATCAGATGCGGACATCGTAGTCTTCCTCAGCTCACTGGATAGTTATGATTCTCTAAAAACCAAGCGCCACCTGTTCGTCCAGGAGATCCGGGAGCAGTTAGAAGCCTGTA**AGCAGAAACATGAGTGGGAAG**TGAACTTTGAGATGTCGAAATGGAAGGCTCCCAGGGTGCTGAGTTTTACCTTGAAATCCA**AGACGCTCGATGAAAGTGTTGAGTTCGATGTCCTTCCCGCCTATAATG**CACTAGGTCAACTGCAGTCTGACTCCACCCCCAGGCCCAAAGTCTACAAGGAGCTCATTGAGCTGTATGCATCGAAGGACATCAAAGGGGGAGAGTTTTCAATCTGTTTTACAGAACTACAGAGAAACTTCATTGAAACCCGGACCACCAAACTCAAGAGTCTGATCCGCCTGATCAAGCACTGGTACAAACAGTATGAAAGGAAGATGAAGCAGAAAGCATCTTTGCCCCCAAAGTACGCCCTGGAGCTGCTCACCGTGTATGCCTGGGAGCAGGGCAGTGGCACAGATGACTTCGACACTGCTGAAGGCTTCCGGACCGTCCTGGACCTGGTTATAAAATACCGGCAGCTCTGCATCTTCTGGACAATCAACTACAATTTCGAAGAGGAACACATGCGGAAGTTCCTACTGACCCAGATCCAGAAAAAGAGGCCTGTGATCCTGGACCCAGCAGAACCCACAGGCGACGTGGGAGGAGGTGACCGCTGGTGTTGGCATCTTCTAGCCGAAGAAGCAAAGGAGTGGCTGTCCTCCCCCTGTTTCCAAGTGGGAAAAAAAGGCCTGGTACAGCCGTGGAAAGTGCCAGTAGTGCAGACCCCAGGAAGCTGTGGAGTTCAGATCTACCCCACTGTGCGTGGGGTTACTCAGTTGGAGTCCATTCAACTCTGGAGTGAAAATTCTGGAAGAAGCTTCTAG

***Protein Kinase R (PKR)***

ATGGCCAATGATACACCAGGTTTCTACATGGACAAACTTAATAAATACCGCCAGAAGCATGGAGTAAACATTACGTATAAAGAACTTAGTACTACAGGACCTCCACATGACAGAAGGTTTACATTTCAAGTTATAATAGATGGGAAGGAATTTCCAGAAGCCGAAGGGAGATCAAAGCAGGAGGCAAAAAATGCTGCTGCTAAATTAGCGGTTGACATGCTTGAGAACAAGGTGGATAGTCACACGGATGCTTCTGAAGAAGGGAACTACATAGGCCTTCTCAATAGTTTCAGGCAGAAGGAAAAGCTGCCTGTAGATTATGAACAGTGTGACCCTGACCCTCAGTTTCCTCAAAGATTTACTTTTAAATGCAAAATTGATCAGATCGTCTATGGTACTGGTTCAGGTGCTACCAAACAGGAGGCGAAGCAGTTGGCTGCCAAAGAAGCATAT**CATAAGCTGGTAGAGAAAGGCC**TAATGAAA**AACGTCCGGGCATCCTTTAGCT**CTGTCATGTCTTCATCCAGTGACACTTTTAGCAGCTCGTCTATGA**CAAGTAACAGTGCTTCCCAGT**CTGCACCAGGAAGTTTTTTCTCAGAGAACTCATTCACGAATGGTCTCAGAGAAAGTAAAAGGAAATCAGAAGTAAAAGTGCCACCTAATGATGTGCTAAGAAATAAATATACCTTGGACACCAGGTTTAACAACGATTTTGAAGACATAGAAGAAATTGGCTCAGGTGGATTTGGCCAAGTTTTCAAAGCAAAACACAAAATTGATGGAAAGATGTATGCTATTAAGCGTGTTAAATATAACACCGAGAAGGCAAAGCGTGAAGTACAAGCGCTGGCAGAACTCAACCACGTCAACATTGTTCAATACCGTATTTGTTGGGAGGGAGAGGACTATGATTATGATCCCGAAGAGAGCATGAGAGATACAAATCGATACAAGACCCGGTGCCTCTTTATTCAAATGGAATTCTGTGATAAAGGAACTCTGGAGCAATGGATAGAAAACAGAAATCGGAGTAAAGTGGACAAAGCTTTGATTTTGGACTTATATGAACAAATAGTGACAGGAGTGGATTATATACATTCGAAAGGTTTAATTCATAGAGATCTTAAGCCAGGTAATATATTTTTAGTAGATGAAAAACACATTAAGATTGGAGACTTTGGCCTTGCAACAGCCCTGGAAAATGATGGAAAACTGCGAACAAAGAGTACAGGAACTGTTCTATACATGAGTCCAGAACAGTCATCTTTAGATACATACGGAAAAGAAGTGGACATCTTTGCTTTGGGCCTTATTCTAGCTGAACTTCTTCACATGTGCTTCTCTGTTTCAGAGAAAATAAAGTTTTTCGGAAGTCTAAGAAAAGGCAACTTTTCCAATGATATATTTGACAACAGAGAAAAAAGCCTCCTGCAGAAATTACTCTCAAAGAAACCCATGGACCGACCTGAGACGGCTGAAATCCTGAAGACTTTGGCTGAATGGAAGAACATCTCGGAGAAAAAAAAAAGGAACACATGCTAG

***Zinc Finger CCCH-Type Antiviral Protein 1 (ZAP)***

ATGATTCAGACGAATATAGCTTCCAAGACTCAAAGGCATGTTGTCAGAAGGCCAGTATTTGTTTCTTCAGAGGATGTGGTGCAGAAGAGAAGAGGTCCAGACCATCAGCCAGTGATGCCCCAGGCAGATGTTCTGACCCTGTATTCTCCTCCCCAGAGGAATACTAGCACTGTTCCTCCTAATGAATATGAGTTTCTAGAACTCCATAGCGAGGATAAAGAATATTTCACAATAAGTGAACTGTTTAAAGCATCCATGAAACATTTCAAGATTGTGAAGATAAAGAGGATATGGAACCAAAAGCTGTGGGACATTTTTGACAGGAAGAAGCAAAAGATGAAAAATAAAAATGAGATGCAACTGTTTAAAGCAGCATGCCATAATCATGTGGATTACATCTGTAAGAACAACTTCGAGTGGATCCTACATGGAAATCAGGAGACCAGATATGGAAAAGGAAATTATTTTGCAAAAGAAGCTATCTGTGCCCACAAGAGTTGTTCATATGACACCAGAGGCACTGTCATGTTCGTAGCCCGAGTCCTGGTTGGAAATGTCATTGAAGGAAATATAACATTCAGTAGCCCTCCTGCGCTCTATGACAGCTGTGTGGACACCAGGCTGAATCCCTCCGTCTTTGTCATTTTCCGGAAAGAACAGATTTACCCAGAATATGTGATTGAGTACGTGGAGTCAGAGAAAGAGAAAGAGAAAGAGAAAAGTTGCGTAATTAGTTAG
