# Supplementary material for: Understanding Host–Virus Interactions: Assessment of Innate Immune Responses in Mastomys natalensis Cells after Arenavirus Infection
Source: Viruses. 2022 Sep 8;14(9):1986. doi: 10.3390/v14091986 (PMC9506377; doi:10.3390/v14091986)
Supplement: Supplementary file 1 [file viruses-14-01986-s001.zip › ISGs Supplementary tables and figures_final.pdf]

Table S1. Master mix set-up for the RT-PCR.

Mix	Gene	Fwd Primer	Rev Primer	Probe	Flourochrome	Quencher
1	**IFIT2**	CTCCTGGCCCTAAACAGTTAC	CCTTGTCTTCACGCTTCATTTG	TCCACCTTCGGTATGGCAACTTTCAG	Cy5	BHQ-2
	**OAS1b**	GAGTGAAGTTTGAGGTCCAGAG	GCTGTAGATGCTAACCTGTCC	TGTGCTTCCAGCCTTTGATGTCCT	JOE	BHQ-1
	**ADAR1**	CTGCCCGTGACTATGACTTAG	TTGCTGATCCAGTTCCCATAG	CCCATATCCCGAAGGCTTTTCTTGAAGT	TxRed	BHQ-2
	**60S**	GATGAATACCAACCCCTCTCG	ATCCCATCCAACACCTTGAG	ACCCTATCACTTCCGAGCCCC	FAM	BHQ-1
2	**ZAP**	AGAGGCACTGTCATGTTCG	GACAAAGACGGAGGGATTCAG	CCCGAGTCCTGGTTGGAAATGTCA	Cy5	BHQ-2
	**OAS2**	AGCAGAAACATGAGTGGGAAG	CATTATAGGCGGGAAGGACATC	CGAACTCAACACTTTCATCGAGCGTCT	JOE	BHQ-1
	**IRF7**	TGGAAAATAGGGAAGAGGTGAC	GTCCCCAGCATCACTAGAAAG	CTCCAAACCCCAAGCCCTCTGC	TxRed	BHQ-2
	**Ppil4**	GTGGGAGACTTACCTGATGC	CTCATCTGTGGTCACTGGATTC	ACACAAACAGCACATTTTCTGGAGGC	FAM	BHQ-1
3	**PKR**	CATAAGCTGGTAGAGAAAGGCC	ACTGGGAAGCACTGTTACTTG	AGCTAAAGGATGCCCGGACGTT	TxRed	BHQ-2
	**Ppil4**	GTGGGAGACTTACCTGATGC	CTCATCTGTGGTCACTGGATTC	ACACAAACAGCACATTTTCTGGAGGC	FAM	BHQ-1
4	**IF27B**	TTGGTTGCTGTGGAGAGTC	CTTCACTGGAACAGGCATTG	AGACATCATCTTGGCTGCTATGGATGC	JOE	BHQ-1
5	**IRF1**	CAAACTTCCGTTGTGCCATG	TGCCCTTGTTCTTGCTCTG	TCCTTCACTTCCTCGATGTCCGGT	JOE	BHQ-1
	**60S**	GATGAATACCAACCCCTCTCG	ATCCCATCCAACACCTTGAG	ACCCTATCACTTCCGAGCCCC	FAM	BHQ-1
6	**ISG20**	ATTATAGAACCCAAGTCAGCGG	AGTCGTGCTTCAGGTCATG	TGTGGCCCTCACCATGTGCT	Cy5	BHQ-2
	**MxA**	CTCAAAAGGAGTGGACTAGCC	TGTCTCCCAATGTTCCTTCTG	CCAGAGATGAGCCAGCACCTGAAT	JOE	BHQ-1
	**MxB**	CTCACTGGTAGAAAGGGAAGTC	CAGGTCAATCAGAGTCAGGTC	CCGGCAATCAAGTTCTGGGCTTT	FAM	BHQ-1

Table S2. Changes in ISG expression of MasKECs after stimulation with IFN-α or IFN-β.

**Gene**	**IFN-α**				**IFN-β**			
**Concen-tration**	**50 U/mL**	**100 U/mL**	**500 U/mL**	**1000 U/mL**	**50 U/mL**	**100 U/mL**	**500 U/mL**	**1000 U/mL**
**ADAR1**	2.0	0.8	1.2	1.3	2.0	2.2	2.2	2.5
**IF27B**	62083.3	29738.4	23542.6	5.7	111.9	129.4	122.3	10.9
**IFIT2**	8.8	4.5	15.5	15.9	4.7	5.6	13.6	15.4
**IRF1***	215.3	96.4	110.6	0.7	1.9	1.5	1.5	1.1
**IRF7**	68.7	33.7	51.9	75.7	102372.1	118873.5	187968.8	263955.0
**ISG20***	474.8	215.8	340.5	#	1.4	1.8	3.2	4.0
**MxA***	357.9	667.1	6216.2	#	5.5	48.3	937.9	2331.9
**MxB***	373.6	995.3	11390.1	14112.2	1.2	115.1	1758.6	3925.9
**OAS1b***	44082.8	41127.4	70105.6	92307.2	149.7	4716.9	48532.9	94513.6
**OAS2***	4797.4	19866.1	9548.6	7063.1	39302.9	55152.0	135798.1	188177.5
**PKR**	10.9	5.0	6.0	6.5	3749773.0	3.8	5.2	6.0
**ZAP**	2.5	1.2	1.5	1.4	1.4	1.8	2.8	3.1

# no Ct values were obtained for neither the experimental nor control-treated cells.

* if no signal Ct value for the controls was obtained due to too low concentrations in unstimulated cells. The previously determined cut-off Ct-values (Table 1) were used for the calculation of the fold change of ISG expression of these genes.

Table S3. Changes in ISG expression of MΦ after stimulation with IFN-α, IFN-β or Poly(I:C).

Gene	IFN-α				IFN-β				Poly(I:C)				
Concen-tration	50 U/mL	100 U/mL	500 U/mL	1000 U/mL	50 U/mL	100 U/mL	500 U/mL	1000 U/mL	2 h	4 h	6.5 h	16 h	24 h
ADAR1	2.4	2.8	2.9	3.2	3.3	2.2	3.3	2.8	3.9	9.8	23.2	12.8	15.4
IF27B*	17518.2	17562.4	25161.2	2909.2	14269.7	13796.7	23815.0	19140.9	#	#	#	195.8	194.3
IFIT2	46.6	65.0	113.8	260.5	60.2	55.4	140.9	189.3	1.7	28.1	287.5	219.7	249.9
IRF1	1.6	1.6	3.1	3.3	2.0	1.6	3.4	3.5	0.9	3.1	8.2	2.6	3.0
IRF7*	56.1	62.0	75.5	466.3	72.5	35.8	100.6	114.2	3.4	32.5	237.7	513.7	625.4
ISG20	1.0	0.4	1.0	0.0	0.1	0.1	0.3	0.2	0.9	1.6	4.1	0.4	0.1
MxA	365.4	348.8	1012.9	2616.1	397.1	391.2	898.2	1263.4	0.2	1.5	165.7	453.7	445.0
MxB	311.3	284.6	795.6	2269.6	108.6	116.8	267.1	373.2	1.7	7.0	779.7	2426.1	2334.9
OAS1b*	541958.8	677514.8	673174.3	159830.9	766420.8	364571.5	539006.2	313659.8	#	#	#	19392.4	24197.9
OAS2*	115.5	127.0	155.5	316.9	141.8	69.2	165.4	170.0	24.0	12.7	1250.0	8157.9	9648.5
PKR	5.5	5.6	1.1	11.1	8.5	7.1	6.1	14239266.9	0.6	2.1	10.7	6.6	6.0
ZAP	2.2	2.3	1.7	1.2	2.7	23346521.0	2.6	2.0	2.1	1.8	6.3	2.1	2.7

# no Ct values were obtained for neither the experimental nor control-treated cells and a calculation of the fold change of expression was not feasible.

* if no signal Ct value for the controls was obtained due to too low concentrations in unstimulated cells. The previously determined cut-off Ct-values (Table 1) were used for the calculation of the fold change of ISG expression of these genes.

Table S4. Changes in ISG expression of MΦ after infection with MORV, MOBV, LASV, RVFV or CHIKV.

Gene	MORV		MOBV		LASV		RVFV	CHIKV
**Time**	**24 h. p. i.**	**48 h. p. i.**	**24 h. p. i.**	**48 h. p. i.**	**24 h. p. i.**	**48 h. p. i.**	**24 h. p. i.**	**24 h. p. i.**
**ADAR1**	1.9	1.4	2.5	1.8	1.2	0.9	6.6	3.2
**IF27B***	#	#	0.7	0.5	1.0	0.5	56705.5	9743.5
**IFIT2**	20.9	15.7	93.2	34.6	12.7	14.0	1516.9	451.8
**IRF1***	1.0	0.7	1012.3	1021.6	172.8	630.8	8.8	1670.3
**IRF7**	35.9	26.6	1161.2	1199.4	5.6	4.6	2010.6	5939.2
**ISG20**	2.5	1.4	#	#	#	#	24.9	3.8
**MxA***	555.4	272.6	5.8	6.6	11.7	3.3	15470.5	32882.6
**MxB***	6823.1	6321.7	784.4	810.4	174.8	159.6	856902.2	46602.7
**OAS1b***	#	#	#	1873.5	#	#	675954.8	83768.2
**OAS2**	907.6	746.2	15907.0	32843.2	31.0	24.5	3071.8	113327.5
**PKR**	2.6	1.8	#	#	#	#	21.7	12.2
**ZAP**	2.0	1.2	3.4	0.4	1.9	1.1	5.6	0.7

# no Ct values were obtained for neither the experimental nor control-treated cells and a calculation of the fold change of expression was not feasible.

* if no signal Ct value for the controls was obtained due to too low concentrations in unstimulated cells. The previously determined cut-off Ct-values (Table 1) were used for the calculation of the fold change of ISG expression of these genes.

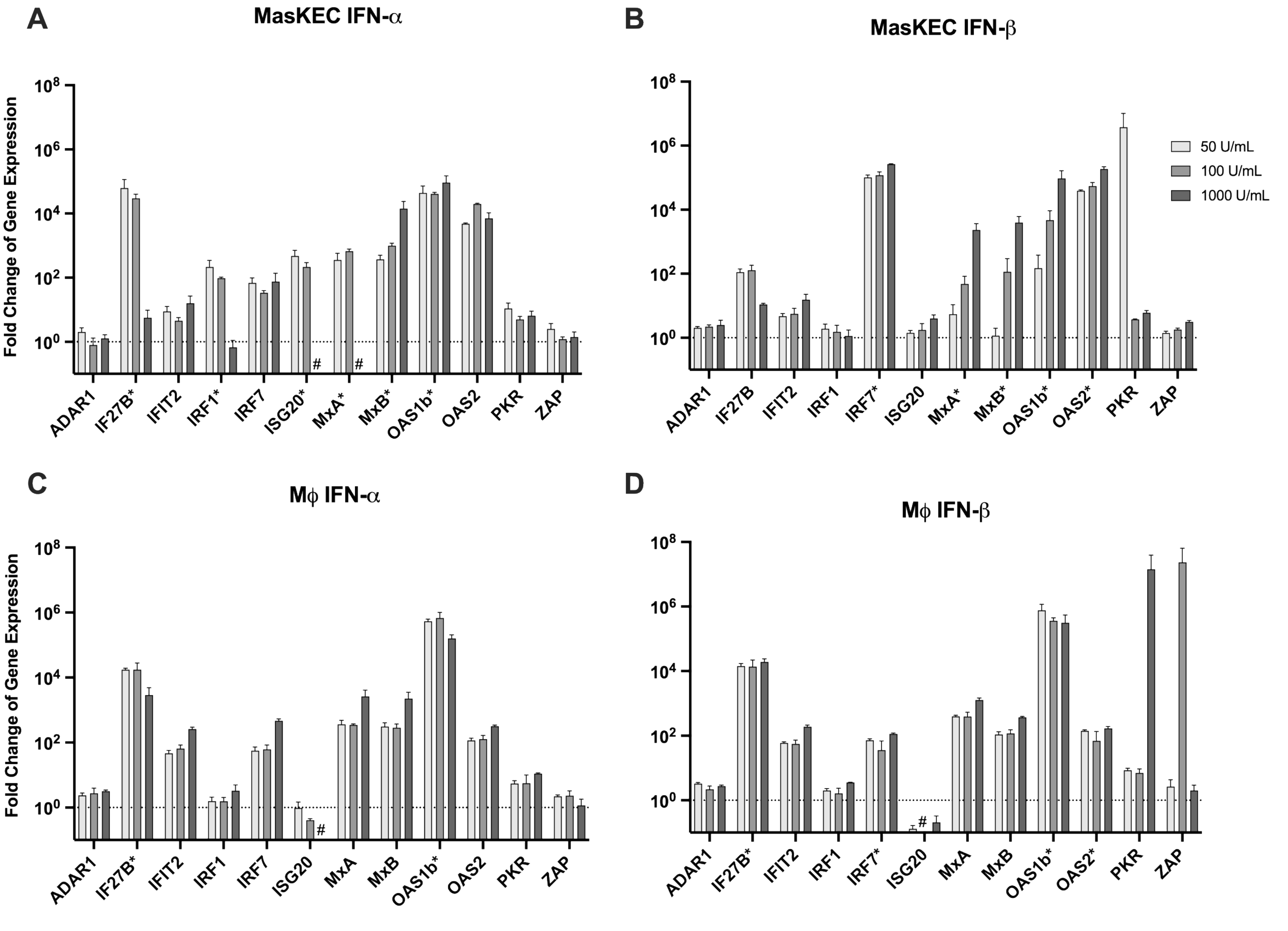
A
MasKEC IFN-α
B
MasKEC IFN-β
50 U/mL
100 U/mL
1000 U/mL
C
Mϕ IFN-α
D
Mϕ IFN-β
Fold Change of Gene Expression
10^8
10^6
10^4
10^2
10^0
#
ADAR1
IF27B*
IFIT2
IRF1*
IRF7
ISG20*
MxA*
MxB*
OAS1b*
OAS2
PKR
ZAP
ADAR1
IF27B
IFIT2
IRF1
IRF7*
ISG20
MxA*
MxB*
OAS1b*
OAS2*
PKR
ZAP
ADAR1
IF27B*
IFIT2
IRF1
IRF7
ISG20
MxA
MxB
OAS1b*
OAS2
PKR
ZAP
ADAR1
IF27B*
IFIT2
IRF1
IRF7*
ISG20
MxA
MxB
OAS1b*
OAS2*
PKR
ZAP

Figure S1. Changes in ISG expression after stimulation with IFN- α or IFN-β. MasKECs (A & B) or MΦ (C & D) were stimulated for 16 h with 50, 100, or 1000 U/mL IFN-*α* (A & C) or IFN-β (B & D). Cells without stimulation were used for later normalization. Cellular RNA was harvested, depleted of DNA and used for RT-PCR analysis of ISG expression levels. For the RT-PCR-based assays, 10 ng of overall extracted RNA was used. Normalization was done with the ΔΔCt method with the housekeeping gene *Ppil4*. Genes were marked with an asterisk (*) if no signal Ct value for the controls was obtained due to too low concentrations in unstimulated cells. The previously determined cut-off Ct-values (Tabe 2) were used for the calculation of the fold change of ISG expression of these genes. (#) No Ct values were obtained for neither the experimental nor control treated cells and a calculation of the fold change of expression was not feasible. The dashed line indicates the ISG level of untreated control cells. The experiment was done in triplicates and depicted are the mean and standard deviation.

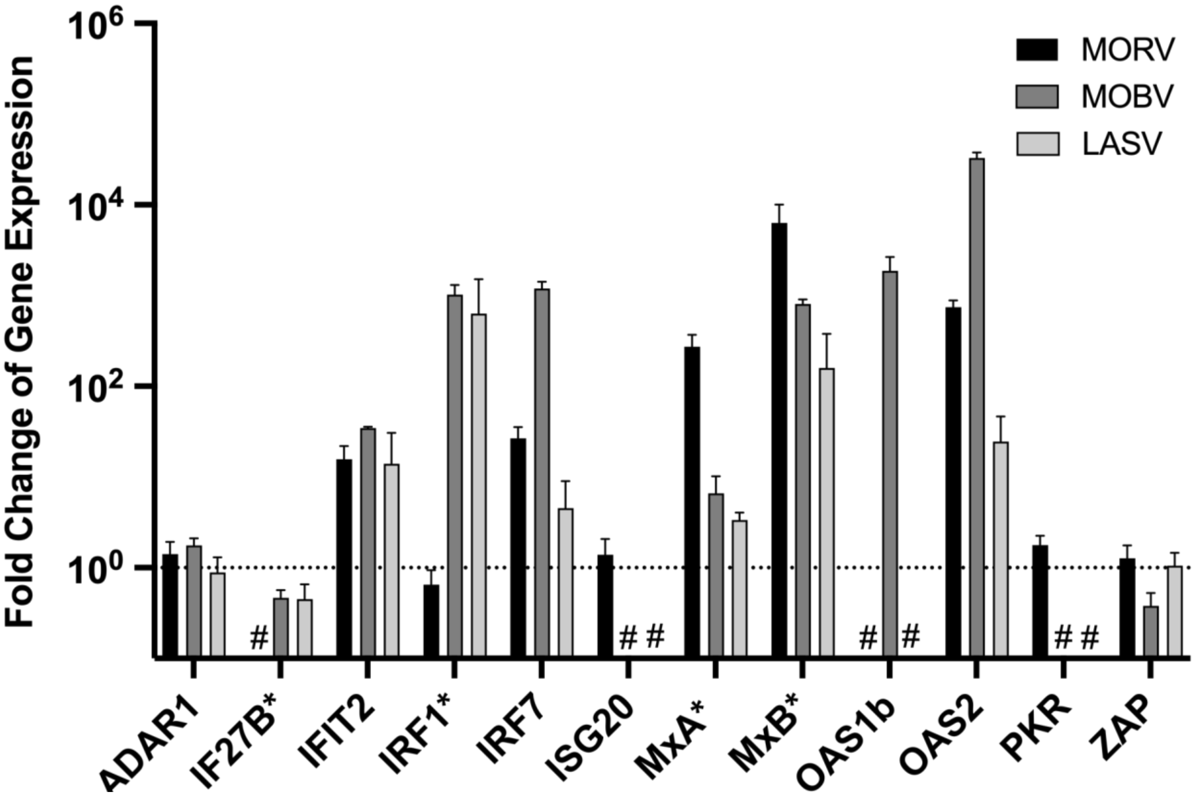

Figure S2. Changes in ISG expression after infection with different arenaviruses. MΦ infected with either MORV. MOBV, or LASV with a MOI of 1 and incubated for 48 h. RNA was isolated and depleted of DNA. For the RT-PCR-based assays, 10 ng of overall extracted RNA was used. Normalization was done with the ΔΔCt method with the housekeeping gene *Ppil4*. Genes were marked with an asterisk (*) if no signal Ct-value for the controls was obtained due to too low concentrations in uninfected cells. The previously determined cut-off Ct-values (Table 2) were used for the calculation of the fold change of ISG expression of these genes. (#) no Ct values were obtained for neither the experimental nor control treated cells and a calculation of the fold change of expression was not feasible. The dashed line indicates the regular ISG level of untreated control cells. The experiment was done in triplicates and depicted are the mean and standard deviation.

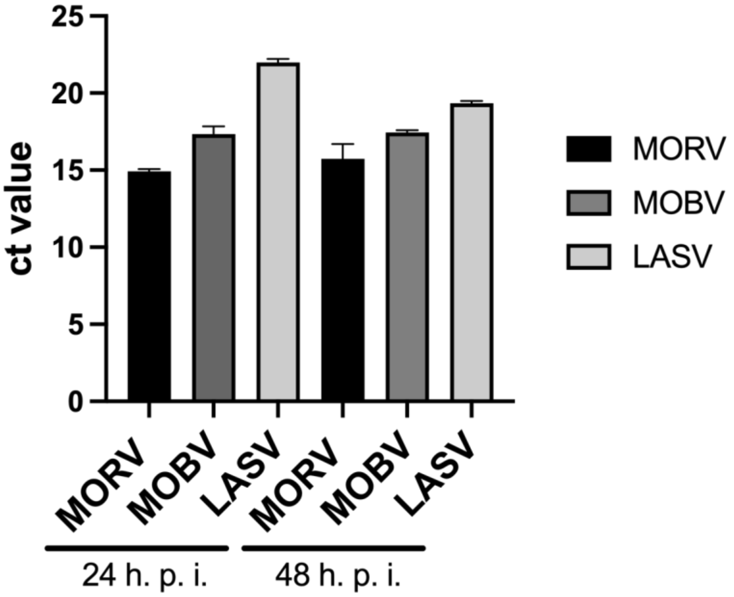

Figure S3. Confirmation of efficient infection with arenaviruses. Mean with standard deviation of all Ct values obtained by using the Altona RealStar® Lassa Virus RT-PCR Kit 2.0. The previously isolated RNA from all infection experiments with arenaviruses was used for the confirmation of efficient infection experiments.
